# Veterinary Department, University of Pennsylvania

**Published:** 1885-01

**Authors:** 


VETERINARY DEPARTMENT, UNIVERSITY OF PENNSYLVANIA.—This institution was formally opened on Oct 2d, 1884, the introductory lecture being delivered by Prof. Huidekoper, Dean of the Faculty. The course of instruction extends over three years, and includes the following subjects:

First Year.—Chemistry, Materia Medica and Pharmacy, Physiology, Histology, Botany, Zoölogy, Veterinary Anatomy and Forging.

Second Year.—Medical Chemistry, Physiology, Therapeutics, General Pathology and Morbid Anatomy, Veterinary Anatomy, Surgical Pathology, Internal Pathology and the Contagious Diseases, Botany, Zoölogy and Practical Farriery.

Third Year.—Therapeutics, General Pathology and Morbid Anatomy, Surgical Pathology and Operative Surgery, Internal Pathology and Cutaneous Diseases, Sanitary Police, Obstetrics and Zoötechnics.

In the second year the student will attend clinics, and will serve as an aid in the hospital. In the third year he will be placed in charge of sick animals and be required to make clinical reports and autopsies. He will also make regular visits to breeding and dairy farms and to slaughter-houses, in order to familiarize himself with the races of animals, the economical means employed in their care and the varieties of butcher-meat. C.

